# Aging, sexual intimacy, and challenges in contemporary India: A qualitative study

**DOI:** 10.3389/fpsyg.2022.946105

**Published:** 2022-09-20

**Authors:** Sanchita Srivastava, Puja Upadhaya

**Affiliations:** ^1^CHRIST (Deemed to Be) University, Delhi-NCR, Ghaziabad, India; ^2^School of Gender and Development Studies, Indira Gandhi National Open University, New Delhi, India

**Keywords:** aging, sexual intimacy, social norm, age norm, family support, social change

## Abstract

An individual's life is shaped by age norms practiced in a particular society. In most societies, there is a deadline for every life event. Sexual intimacy is an essential part of every individual. However, sexual intimacy seems appropriate for young individuals, and middle-aged and older are considered asexual. Those who share sexual intimacy at a later age have to face the consequences for this age-inappropriate behavior in society. This study analyses “Badhaai Ho” film to explore the consequences of sharing sexual intimacy by middle-aged heterosexual couples in their 50s as it is forbidden by prevalent social norms. This study also explores the role of family in dealing with the repercussions of actions against the prescribed social norms. Thematic analysis suggests that society has a predefined age-bound box for individuals with different age categories. The middle-aged couple suffers various consequences for breaking the prescribed age-bound box. The role of the family is found to be crucial in mending the box by replacing it with an updated version. There are also gender differences in attitude toward sexual intimacy. Implications of this study can be utilized to explore the pathway of social change in existing social (age) norms in any society.

## Introduction

“*With ‘Badhaai Ho,' I tried to normalize the sexual desire that our parents can have, and there's nothing wrong in it” —* Ayushmann Khurrana (National Herald, 2020)

In the previous excerpt, Ayushmann Khurrana, an Indian actor discussing his recent Bollywood film “Badhaai Ho,” put forward a dialogue about sexual intimacy between aging heterosexual couples as “normal” and hence should be accepted in society. This film is classified in the comedy genre with a subject that seems forbidden or subject of public embarrassment or shame [sexual intimacy between the middle-aged couple and decision to become parent, despite having two grown-up children]. The filmmakers decided to delve into a serious subject for creating laughter among their audiences. Perhaps, they took this subject since it is not commonly welcome in contemporary Indian society. It makes room for turning this embarrassing and awkward subject into comedy with a positive message. Sexual intimacy, romance, and childbirth among aging heterosexual couples are not commonly shown in contemporary Bollywood. Aging couples are generally portrayed as parents and caretakers, and romance and sexual intimacy are only reserved for young people. Like any other societies, sexual intimacy and aging norms in Indian society are inspired by religious/cultural beliefs and normative practices prevalent in the society. Laumann et al. (2006) highlighted that in Asian countries, subjective sexual wellbeing of older adults is at a low level, and it might be due to the culture of the society. Public discussion on sexual intimacy and sexuality is dependent on societal practices and prevailing norms. Studies on sexuality at a later age in the Indian context suggest the presence of sexual practices among middle-aged heterosexual couples (Kalra et al., 2011; Dhingra et al., 2016); however, public discussion on the same is mostly absent to avoid the negative societal attitudinal reaction. Conversely, sexual intimacy is accepted and promoted among young heterosexual couples in most societies including India as these young couples reproduce and take family responsibility as adults, followed by age deadlines set by any given society (Neugarten et al., 1965) or religious/cultural doctrine such as ashrams in Hindu religion (Olivelle, 1993; Saraswathi et al., 2011). Despite such a positive attitude toward young couples' sexual intimacy, the open dialogue about sex, sexual needs, and sexuality is missing, resulting in a societal taboo about such topics in India (Dhingra et al., 2016). In such a scenario, open discussion or disclosure about continued sexual intimacy among middle-aged and old aged couples is challenging. Rao et al. (2015) conducted a study on the Indian population and found that people carry a popular myth that sexual activity is only meant for procreation, and not for pleasure, and indulging in sexual act at a later age is perceived as shameful and morally inappropriate behavior.

Although various doctrines of religion and spirituality have inspired our lives, everyday life is inspired and guided by common practices, which are often labeled as “societal norms.” These norms serve as a codebook to plan our lives and fit ourselves in the existing model of society. Sexual intimacy and age do not necessarily share an inverse relationship among couples. Sharing sexual intimacy at a later age supports maintaining psychological and sexual health among aging couples (Hillman, 2012; Štulhofer et al., 2018; Souza et al., 2021; Traeen et al., 2021). Although healthy sexual practices and sexual intimacy among aging couples bring positive outcomes, many societies mostly perceive this as negative (Rowntree, 2014). Various reasons have been identified for such negative perception, such as aging couples are considered “asexual” due to social norms and religious beliefs (Le Gall et al., 2002 Gott and Hinchliff, 2003; DeLamater, 2012). The available literature in this area has primarily focused on the biological aspect of sexuality at a later age (Lindau et al., 2007). Few studies explored people's attitudes toward sexual intimacy at a later age (Haesler et al., 2016; Lee et al., 2016). Why does age play an important role in determining sexual intimacy between couples? The possible answer could be age norms that serve as a reference point for individuals in every society to decide suitable timing for education, marriage, childbearing, rearing, etc. Thus, age seems to be one of the essential aspects of our social structure that organizes our lives in various categories.

Neugarten et al. (1965) had studied the phenomenon of “*age norms*” and conducted a study on age norms and age constraints imposed on individuals as a part of their socialization process in American society (Neugarten et al., 1965; Neugarten, 1969, 1981). They emphasized “age-appropriate behavior” that provides an individual with a “*prescriptive timetable for the ordering of the major life events: a time in the life span when men and women are expected to marry, a time to raise children, a time to retire*” (Neugarten et al., 1965, p. 711). This defined timetable is followed by most of the members of every society. The prescriptive timetable provides a clear vision or a “mental map” to every member of the society to prepare for their significant life transitions in advance (Hagestad and Neugarten, 1985). Additionally, individuals also learn through socialization proscriptions for various age categories. These informal prescriptions and proscriptions of age norms are exercised with social sanctions. Those individuals who fail to follow the prescribed age norms experience various consequences, ranging from being ridiculed for their behavior to complete social exclusion (Neugarten, 1979; Settersten and Hägestad, 1996a,b; Wrosch and Freund, 2001; Settersten, 2002, 2003).

Settersten and Hägestad (1996a,b) investigated “age deadlines” provided by their participants regarding family, education, and work-related significant life transitions for men and women. Life transitions have two major categories: “Family transitions and educational/work transitions.” The major family-related life transitions were *leaving home, returning home, marrying, entering parenthood, complete childbearing, and entering grandparenthood*. Additionally, education and work-related significant life transitions mentioned by the study participants were *full-time exit schooling, enter full-time work, settle on career/job, the peak of work career, and retire*. The majority of the participants in their study supported these life transitions with specific age limits. Additionally, gender differences were observed in following age deadlines and life transitions in a given society (Zepelin et al., 1987; Settersten and Hägestad, 1996a,b; Settersten, 1997).

The age-bound categories and life transitions are found to be practiced universally. For example, in India, the culture and social norms are influenced by specific worldviews, social practices, and religious doctrines such as Hinduism (Olivelle, 1993). Hinduism guides toward the path of “*moksha*,” an ultimate goal of human existence. The path of “moksha” is achieved only after completing four stages or ashrams: *bhramacharya, grihastiya, vanaprastha*, and *sanyas* ashrams (Olivelle, 1993; Saraswathi et al., 2011). Every ashram is age-bound and attached to specific duties and responsibilities. Till the age of 24 years, one lives in bhramacharya ashram to attain knowledge and complete education. From the age of 24–48 years, one lives in grihastiya ashram and fulfills all the worldly duties related to wealth and pleasure. Then comes the vanaprastha ashram from the age of 48–72 years, where an individual practices detachment, handovers the family and worldly responsibilities to the next generation, and continues the path of freedom or moksha. The last ashram (from 72 years to death) is dedicated to achieving liberation or moksha. In Indian society, individuals are not completely independent from family to follow these ashrams; moreover, the family holds an essential place in smooth functioning and practicing such ashrams timely due to collectivistic culture.

Furthermore, the family also plays an important role in an individual's life to provide support and resources during significant life transitions. Lack of such support may affect an individual's wellbeing and development (Uhlenberg and Mueller, 2003). Support from family members and spouse increases the self-efficacy of the elderly (Patrão et al., 2018a,b). Increased self-efficacy among the elderly helps them maintain their mental and psychological health. The availability of such social support and resources is dependent on an individual's “on-time” and “off-time” age-related behaviors (Neugarten and Hagestad, 1976). Recently, Settersten and Thogmartin (2018, p. 360) highlighted the “*social aspects of life transitions*” and advocated that “*life transitions are rarely individual: they are relational…Family and other relationships can be key sources of support for transitions but also create risks*.” They emphasized the interpersonal side of the life transitions as family and individuals influence each other.

The present study aimed to explore the existing age norms, age-appropriate behaviors, and consequences of off-time sexual intimacy in contemporary Indian society. Past studies on age norms and sexual intimacy have been examined in many countries (Lagana and Maciel, 2010; Yan et al., 2011; Agunbiade and Ayotunde, 2012). However, there is a dearth of research in studying their application in the Indian context. Studies on age norms and life transitions also explored gender differences in “on-time” life transitions; however, gender differences in experiencing the consequences of “off-time” life transitions have remained less explored. The role of social support has also been studied only in the case of “on-time” life transition events. Still, there is lack of studies of the cases where family provides support to individuals', despite being targeted as violators of age norms and practices. The present study had attempted to fill these gaps by analyzing a recent and popular Indian Bollywood film, “Badhaai Ho.”

Choosing film for this qualitative study is motivated by the multiple functions fulfilled by film analysis (Mikos, 2014). The film is always seen as a potential form of media that represents society and, at the same time, communicates with it. It will undermine a film's potential if it is only perceived as a mere source of entertainment, rather than as a reflection of our society and the agent of change (Teary, 2012). In addition to this, a film serves as a powerful tool to redefine existing cultural traditions with the social message to influence masses. A film also allows us to get perspectives and realities of many lives that we miss acknowledging due to lack of opportunity and time in this busy world. Hence, through a film, we get a closer look at many topics through storytelling, ranging from mundane to exceptional.

### Film summary

This film is based on a middle-class Hindu family (Kaushiks) who lives in a government quarter in Lodhi colony, Delhi, India. In this film, three generations live together (grandparents, parents, and children). The main characters (Kaushik family members) of the film are described in Table 1. The film revolves around two main characters: Jitender/Jeetu and Priyamvada. They are in middle age and accidentally expect a child due to sexual intimacy. The couple in their 50s struggle with this unexpected pregnancy and experience stress, anxiety, embarrassment, mocking, and even rejection by their family and immediate society (including extended family, neighbors, friends, and workplace). The story focuses on Amma and Nakul's strong negative reaction toward the pregnancy against the backdrop of continued sexual intimacy between these middle-aged couples. Renee (Nakul's girlfriend) is shown in an important role in breaking the ice for Nakul to understand his parents' continued sexual intimacy and decision to keep their unexpected child, despite social consequences. The negative reaction of the society makes it hard for the couple to accept their upcoming child. However, after the support received by Amma and Nakul, the couple gets free from the burden of guilt for putting the reputation of their family at stake. As a result, they happily accept and openly express their joy for their upcoming child.

**Table 1:** Main characteristics of the film.

**Main character (played by)**	**Role description**
Amma (Surekha Sikri)	Oldest member in the family and Jitender/Jeetu's mother
Jitender/Jeetu Kaushik (Gajraj Rao)	Owner of the house
Priyamvada Kaushik (Neena Gupta)	Jitender Kaushik's wife
Nakul Kaushik (Ayushmann Khurrana)	Elder son (25 year old) of Jitender and Priyamvada Kaushik
Gular (Shardul Rana)	Younger son of Jitender and Priyamvada Kaushik
Renee (Sanya Malhotra)	Nakul's girlfriend and colleague

## Methods

To fulfill the aim of the present study, we have chosen a Hindi film, “Badhaai Ho” directed by Amit Ravindernath Sharma. This film was released on the 18th of October 2018 in Hindi in India. This film lasts 123 min. A few points are considered while choosing such a film as a subject of study. First, the subject matter of this film is middle-aged sexual intimacy, pregnancy, and its repercussions in contemporary Indian society, and the role of the family in dealing with an issue challenging the existing cultural norms, expectations, and popular practices. The second salient feature of the selection was the wider reach of this film to the audiences of India and worldwide. This film was marked as a commercial success, with the box office earning more than 200 crore rupees worldwide. Third, this film has also won many awards, such as Filmfare Awards, Star Screen Awards, and other awards platforms, and positive reviews from the audience and film critics in India.

This study had chosen for qualitative “thematic analysis” introduced by Braun and Clarke (2006, 2013, 2019) to analyze the film. This method includes six steps: “familiarization” with the data. This step involves a thorough understanding, preparation, and knowledge of the whole data by reading or watching it again and again. After getting the initial understanding of the data, one moves to the second step of “generating initial codes.” This step allows the researcher to break the data into small initial codes or chunks and identify similar and meaningful patterns from the whole data set by using an inductive or deductive approach. The third step includes the process of constructing or searching for the “candidate themes” from the initial coding. After developing the “candidate themes,” the fourth and fifth steps involve “reviewing and defining themes” from the data. The final step is to present the whole data analysis in themes and ensure that these themes are close to the original data and research questions. Braun et al. (2019) advocated for the reflexive thematic analysis method. They suggested that in this analysis, “the researcher is a *storyteller*, actively engaged in interpreting data through the lens of their cultural membership and social positioning, their theoretical assumptions and ideological commitments, as well as their scholarly knowledge” (pp. 848–849).

Using the ideology and standpoint from the reflexive thematic analysis developed by Braun and Clarke (2006, 2013, 2019), data had been analyzed in the present study. Since analyzing a film involves visual and audio data, the audio and visuals of each scene of the whole film were transcribed. Each scene was interpreted as a viewer through narration, including facial expression and the story enacted in the scene. The film was watched several times by both the researchers to develop familiarity with it. Then, the scene-wise interpretation of the whole film was developed. To draw themes based on a “shared meaning-based pattern,” the entire data were reviewed thoroughly to develop a certain level of depth during the initial coding process. Each scene was then analyzed and given initial codes by one of the researcher and reviewed by the other. However, coding was carried out in an open-ended way and progressed. Later, these initial codes were collated into “candidate themes” and again reviewed from the aim of the present study. During this process, many subthemes were developed. Successful subthemes were reviewed again, and their potential to contribute to the subject of the study was assessed. The meaningfulness, necessity, and capability in contributing to the storytelling were the major criteria of keeping or dropping a theme in the analysis process. The remaining themes were further reviewed and defined by ensuring that they contribute to the main concepts or phenomena studied. The inductive analysis followed reflexive thematic analysis; therefore, both the researchers intended to bring diversity, richness, and multiple perspectives during the data analysis process as collaborators. A constant discussion between both researchers during the coding and development of themes was encouraged to bring unique and common perspectives around the central concept of the study (Braun and Clarke, 2019). The final themes are reported and discussed in the next section.

## Results

A total of three major themes were developed using thematic analysis. Each theme and related subthemes are discussed as follows:

1. Age-bound box.
2. Age-bound box was accidently broken.
  2.1 Penalty for the culprit.
  2.2 Toll for the family.
  2.3 Women are more blamed than men.

3. Family came forward and fixed the broken box.
  3.1 Modification done to self.
  3.2 Altered the broken box with an updated one.

1. Age-bound box

Right from the opening scene, the film sets the tone of the age-bound box, which we define as age categories with certain expectations and behaviors of the individual. Several scenes of the film reflect that three generations with specific age groups (old age, middle age, and young age) share a unique age-bound box. For instance, the grandmother is shown and referred to as involved in religious activities such as performing religious prayers with extra care, counting her religious band on several occasions, and participating in religious gatherings every evening. On the other hand, two children are shown in the family: the elder son as a young adult and the younger son in his teenage. The elder son Nakul is shown successfully managing his life with a good position in his job. He is also shown approaching his relationship with his girlfriend to a future marriage prospect. The other characters (as young adults) discuss their romantic relationships. The younger son, Gular, is shown enjoying his youth with a more care-free attitude toward life, especially regarding his studies.

On the other hand, parents are shown to take care of their family and balance their mother and children's needs. Even the other characters in their middle age discussed the daily hassles of life. However, this middle-aged (Jitender and Priyamvada) couple shares sexual intimacy, which later results in an unexpected pregnancy and leads the couple to suffer from age-inappropriate behavior.

In one scene, Amma reacts to the off-time pregnancy,

“*From now onwards, who so ever will see your sons, would be saying…. here are Jitender's sons going, while carrying Jitender's son (newborn) in their hand. In the age of having one's own child, would Nakul help in raising a newborn sibling?”*

She also blames Jitender and Priyamvada for not following the age-appropriate behavior, as she says to her son,

“*I used to say, watch 'sanskar tv' (religious and spirituality related TV channel), and you always preferred to watch news channel (referring news channel as a substitute to engagement in the worldly affair) and now see the result (off-time pregnancy)”*

She further questions Priyamvada's moral behavior after learning about her pregnancy:

“*She, the daughter of 'baid sahab,' did not experience any shame (she is referring to the sexual intimacy with her husband), her children have grown up and started doing the job, even now she puts lipstick.”*

On the other hand, Nakul also reacts negatively to the pregnancy and says to his girlfriend,

“*In the age when parents start preparing for their son's wedding, these people (his parents) are preparing for 'Sarbala' too. (sarbala is a boy child dressed similar to the groom and sits next to the groom during the wedding procession in many Indian Hindu marriages as part of the tradition).”*

Furthermore, during his intimate moment with his girlfriend, Nakul again thinks about his parents' sexual intimacy and questions the appropriateness of such behavior at their age. He says,

“*You just tell…. is this a thing (referring sexual intimacy), in which mummy-papa (parents) should get engaged with?”*

2. Age-bound box was accidentally broken

The age-bound box suggests that the individual's behavior should be specific to their age norms in a given society. However, the couple engages in sexual intimacy at their middle age, resulting in an off-time pregnancy. This pregnancy puts them in a difficult position regarding their family and society. The previous theme mentioned the expectations and norms set for middle-aged people by showing rejection toward the couple's intimacy. Furthermore, the reactions of others toward the middle-aged couple are found to be gendered. Three subthemes—penalty for the culprit, the toll for the family, and women are blamed more than men—are discussed separately.

2.1 Penalty for the culprit

Bond and sharing between family members is a key for family welfare. However, when the family environment becomes stressful, each family member gets affected. Those individuals who bring the stressful event to the family and cause others stress may experience dual stress. Similarly, this couple experiences the burden of unhappiness to their family and has conflicting emotions about getting pregnant with another child. Their off-time pregnancy affected the couple's relationship with others in the family and their relationship. Several film scenes show that this couple experiences negative emotions, stress, and embarrassment for continuing sexual intimacy at middle age, which gets open to public scrutiny after pregnancy news.

The pleasant and loving relationship between the couple gets affected after they are rejected by their family, especially Amma, the oldest family member who gets extremely angry with the pregnancy news and questions their intimacy at middle age. Afterwards, Priyamvada blames Jeetu for such accidental pregnancy and warns him to stop being romantic and intimate with her in the future:

“*When Jeetu is trying to pacify Priyamvada after his mother blamed her for this pregnancy, he says, I request you to forget your anger because it doesn't suit your personality. Priyamvada responds; first, you should stop talking about such romantic talks as this pregnancy is the result of it (referring to his romantic talks which persuaded her to get intimate with him).”*

In another scene, Priyamvada warns Jeetu to dare bolt their bedroom at night as Amma questions their privacy. She says,

“*From now onwards, don't you ever try to bolt our room.”*

In one scene, the family environment worsens when the elder son confronts his father, questions their tempered social image due to the off-time pregnancy, and refuses to be part of the family function to avoid becoming the subject of public ridicule. It creates extreme stress in the family and leaves Jeetu angry and Priyamvada hurt and helpless as her family members avoid her. The stressed family dynamics become more evident to others (relatives) when Priyamvada and Jeetu attend a family wedding occasion without their children. Even neighbors and relatives also mock and judge Jeetu and Priyamvada on morality.

2.2 Toll for the family

Amma experiences stress and worries her grandsons, especially toward Nakul, who is then in marriageable age: it must be so embarrassing for him to have a new sibling. She also expresses concern for the family reputation as their intimacy is becoming known to everyone for mocking. In several scenes, Amma expresses anger toward Jeetu and Priyamvada and experiences pain by seeing that the healthy family relationships are getting tense and negative. Similarly, Gular is also shown angry and tense after the pregnancy news. Nakul even scolds him for being responsible for such mishap (pregnancy) as Nakul slaps him, and then Nakul says:

“*You were in a hurry to get your room, has it now? Couldn't you sleep a little longer in mummy papa's room?”*

Gular is even shamed and ridiculed by some schoolmates for his parents' intimacy, which turns into a fight, and those boys beat him. Later, after a school lecture on sex education, Gular also experiences guilt as if he is responsible for his parents' pregnancy because he takes away a pack of condoms from his parents' bedroom out of curiosity.

Even Nakul also gets affected by the pregnancy news as first he is shocked at how this could be possible at this age: he never thought that his parents could continue their intimacy even in middle age. After that, he feels embarrassed to face others with this news. He avoids his girlfriend for a long time as he could not muster the courage to associate “pregnancy” with his mother. In one scene, Renee, who has been avoided by Nakul for quite a long, decides to visit his place and encounters him directly. She expresses anger, worry, and awkwardness to Nakul for ignoring her without any fault from her side.

Nakul's neighborhood friends also attempts to make fun of him due to his parents' pregnancy. As a result, Nakul avoids his friends too: acute stress and embarrassment are visible in many scenes, where he tries to find solace in alcohol and isolation. His friends keep coming to Nakul's home to encounter him since he has constantly avoided them. They keep on messaging him and try to stick a sticker (an indicator of the baby) on his car to make fun of him through the pregnancy news.

2.3 Women are blamed more than men

Another important theme was mentioned regarding the gender differences toward the off-time pregnancy and sexual intimacy shown in the film. The first difference is seen when the family doctors (male and female) found Priyamvada pregnant in her 50s. The reaction of both doctors toward Jeetu and Priyamvada is different. At first, the female doctor expresses disapproval toward Jeetu as she blames his irresponsible behavior (which caused pregnancy). On the other hand, the male doctor expresses excitement, joy, and surprise toward Jeetu (as if he was elated by Jeetu's man potential). Both doctors show concern toward Priyamvada as she has to bear the consequences of their unplanned intimacy.

On the contrary, when Amma learns about their pregnancy, she is shocked and initially blames Jeetu and Priyamvada for their shameful deed. However, later, she blames only Priyamvada for this off-time pregnancy. She scolds Priyamvada even harsher and points toward her makeup: what is the purpose of applying makeup at this age? These comments of Amma toward Priyamvada and Jeetu reflect that she blames Priyamvada for the pregnancy as it was her job to control men's desire, and it was her makeup and privacy she allowed to Jeetu that might have provoked him for sex. It makes Priyamvada angry and sad as she could not tolerate such humiliation and allegations from her mother-in-law. Later, Priyamvada shares her pain with Jeetu and says,

“*She gave a character certificate to her son; however, she assassinated my character, and why had she pulled my father's name in this?”*

Even when this couple attends a family function, they are shown to be treated differently about their off-time pregnancy. It was interesting to see how male and female relatives and others had treated Priyamvada and Jeetu differently on that family occasion. The men (relatives and friends) show a positive reaction toward Jeetu after learning about his upcoming child. He is greeted by and even given extra attention. His old friend Chawla seeks his expert advice in helping out his own son's sexual performance-related issues, indirectly referring to Jeetu's masculinity at middle age.

During this family function, Jeetu is shaving his beard and leaving his mustache to grow in a morning scene. He also seems to admire his mustache. Priyamvada gets very upset and angry with Jeetu as she says,

“*Now you are attempting to grow your mustache as a sign of masculinity to earn respect in the male fraternity, whereas I am constantly hiding my pregnant belly from others (referring to avoid social humiliation)”*

This conversation reflects two things: first, the mustache is seen as a symbol of masculinity and male prowess; second, the gender differences in reacting toward pregnancy at middle age. Jeetu is happy because this pregnancy proved his masculinity and gives him the opportunity to be accepted and appreciated among his male fraternity. However, on the other hand, most women (relatives and others) at the wedding are engaged in mocking and humiliating Priyamvada by their words and actions. This witnesses the reservation to this off-time pregnancy among the female fraternity. Her sisters-in-law blames her for becoming the laughing stock during the family function (wedding) and putting their family reputation at stake, making them experience shame and embarrassment in front of relatives and guests. They say to Priyamvada,

“*You were the center of attention (middle-aged pregnancy was the much-discussed topic during the wedding) during the whole wedding, see Priyam (Priyamvada) neither we are not in a position to interrupt you for doing something, nor you would like to be questioned either? However, you should have given thought to our family reputation as a whole.” (said by elder sister-in-law aka wife of Jeetu's elder brother)*

“*Bhabhi [referring to elder sister-in-law], Shanu's mother-in-law also asked me about Priyam [referring her pregnancy], I felt too ashamed to tell her [confirming priyamvada's pregnancy].” (said by younger sister-in-law aka younger sister of Jeetu)*

“*You should have thought of how the family would be affected by this…what society would say…and also about your sons, especially Nakul…. See he was too embarrassed to attend his cousin's wedding (said by elder sister-in-law aka wife of Jeetu's elder brother).”*

“*And what will your children learn from your behavior [referring intimacy and pregnancy at middle age when people stop being intimate and prepare for their children's wedding].” (said by younger sister-in-law aka younger sister of Jeetu)*

3. Family came forward and fixed the broken box

Another significant theme emerged with the role of the family as a “rescuer” who came forward, especially when they realized Priyamvada had suffered a lot. They have to intervene and protect her self-respect and social image to avoid severe harm. However, this process of changing their position from “destroyer” to “saviour” needed conscious efforts to understand the situation and evaluate whether the damage that happened to this middle-aged couple was fair or not. The family reassesses the grounds of punishing the couple and decides to change their approach toward this off-time pregnancy and redefine the age box preventing this middle-aged couple from celebrating parenthood. Here, two subthemes are discussed separately: modification done to the self and altered the broken age box with an updated one.

3.1 Modification done to the self

To take a stand on the wrong happening with Jeetu and Priyamvada, the family has an important duty to protect their members from harm. First, it required them (Nakul, Gular, and Amma) to understand the factors that made them against their family members. Nakul and Gular were embarrassed due to their parents' pregnancy as people were laughing at them and sometimes questioned their parents' character on morality grounds. However, after a long battle within themselves, they realize that they love their parents and cannot allow others to disrespect them. Nakul's girlfriend helps him in realizing that parents are first sharing a relationship of a married couple with a caring and intimate bond with each other and that he should respect. Another important force behind Nakul's and Gular's decision to stand for their parents is influenced by realizing that their parents are questioned on their morality and that at the end, they are their parents who gave them birth. If they wish to keep their baby due to their intimacy, they should have a right to do so. Whatever relationship their parents share is private, and they should respect it. They should first accept the upcoming child as part of their family and face others with the confidence to consider this issue with openness. In one scene, Nakul and Gular discuss that nobody has a right to disrespect their parents and that it is high time to take a stand. As Nakul says to Gular,

“*Enough is enough…my brother Gular…Now onwards, we wouldn't hide anymore.”*

In another scene, Nakul, avoiding his friends due to his parents' pregnancy, finally decides to face them. They start teasing him for getting privileged to become a brother second time at this age. He takes it positively, stands firm, and responds even in a naughtier way, which showed his acceptance of his parents' sexual intimacy and the pride he felt for them.

On the other hand, Amma—who initially scolded Jeetu, became extremely harsh for Priyamvada and questioned her character, and talked about their selfishness in indulging in worldly pleasure—after slowly analyzing the reason for her anger and weighing the righteousness of the pain and hurt Priyamvada has been experiencing from the day she learnt about her off-time pregnancy, comes forward and replies to family members and relatives that moral and cultural values teach us so many other good things. The intimacy between couples should be socially accepted, and they should not be publicly ridiculed even if they continued such proximity after stepping into middle age. As she says,

“*Is it morally wrong for married couples to share intimacy and love with each other?”*

3.2 Altered the broken box with an updated one

Another essential theme emerged as the family played an important role in questioning the existing system and replacing the old norm with the newer one if it victimized the innocent. It was quite evident through the previous theme that the family members of this middle-aged couple (Jeetu and Priyamvada) had attempted their best to adapt according to the need and to acknowledge the off-time pregnancy confidently. This massive step to stand for their family members and support them in feeling accepted and positive might influence society. The preparation of a “baby shower” for Priyamvada could be an example of setting new norms for pregnancy age and middle-aged couples' intimacy acceptance.

For example, Nakul first felt so embarrassed and hesitant to even say that his mother was pregnant. Later, after self-acceptance, he takes his mother to the hospital for a routine check-up. He is encountered by an adult couple who is waiting for their turn. They tell Nakul that they are a little late in the family planning and, assuming that he is also waiting for his pregnant wife, praises him for being on time. As soon as they finish talking, Priyamvada comes out of the doctor's cabin and asks Nakul to leave with her. When the couple sees this, the husband corrects himself and assures his wife they are not too late for becoming a parent compared to Nakul's mother. It reflects that norms could be redefined when ample examples show acceptance and happiness within the family.

After successfully delivering a girl child in another important scene at the hospital, Priyamvada asks Nakul to capture this beautiful, emotional moment through a selfie. While Nakul was taking a selfie, Jeetu's mother suggests that Jeetu take this opportunity to be in the hospital and get himself operated on as a precaution to avoid being in the same situation next year. It made everyone laugh as she just cracked a joke out of this situation, but if we analyze her statement, it reflects that now she accepted Priyamvada and Jeetu's sexual intimacy. In addition to this, in the final scene of the film shows the ultimate acceptance by the family members, relatives, and neighbors who took part in Nakul's engagement, who earlier showed reservation, for Jeetu and Priyamvada's intimate relationship and pregnancy. It reflects an important message: if a family comes together and remains united under challenging times, it can challenge and face adverse reactions of society. Family acceptance and support had turned the off-time pregnancy into the most desired event of their life. This also sets an example that when the family accepts, nothing remains forbidden.

## Discussion

“*…a middle-aged couple who decide to have another child are criticized because of the presumed embarrassment to their adolescent and married children…”* (Neugarten et al., 1965, p. 712).

This quote represents the core area of the present study, an example of “age-inappropriate” behavior. This age-inappropriate behavior is studied through the Bollywood film *Badhaai Ho*. The literature on age norms and life transitions suggests its significant impact on determining an individual's life course. However, when the individual deviates from the “prescribed timetable” or “acts off-time,” it would immediately be perceived as a threat to the social order of society *via* various norms. This would potentially lead the individual and their family to be exposed to multiple consequences. The present study has found three important themes: “age-bound box, age-bound box accidentally broken, and family came forward and fixed the broken box.” These three themes help understand the complex relationship between “age norms” and “life transitions.”

The age-bound box theme showed that in Indian society, people are categorized into different age categories. These categories are attached to certain expected behaviors linked to specific life transitions (Neugarten et al., 1965; Settersten and Hägestad, 1996a). The main characters of the film showed the following age-specific roles and activities that resemble the “ashram system” of Hindu worldviews (Saraswathi et al., 2011). For example, Amma is mainly interested in religious activities according to the “sanyas ashram.” By contrast, Jeetu and Priyamvada are shown managing the needs of their family members and constantly guided to be ready to leave the “grihastiya ashram” and stop focusing on worldly pleasure (such as money and sex). However, later, they were criticized and rejected because of their sexual intimacy.

The “age-bound box was accidentally broken” theme showed that Jeetu and Priyamvada were labeled for having age-inappropriate behavior resulting in off-time pregnancy. The age-bound box represents specific behavior and goals to be achieved according to their age category. They experienced various negative consequences and were charged guilty against the “prescribed timetable” (Ménard et al., 2015). Banerjee and Rao (2022) in their study on perception of sexuality at later life found that there are common myths in Indian society as “*older people are asexual, people should not discuss sex at later life, spirituality and sexuality are antagonistic in older people, one needs to be ashamed and guilty if sexual thoughts occur in old age*.” Due to these types of stereotypic beliefs, people at a later age could not muster the courage to express their desire, sexual expression, and interest. Past studies confirm that family transitions adhere to strict deadlines for each event, especially the childbearing process (Zepelin et al., 1987; Peterson, 1996; Settersten and Hägestad, 1996a). In Indian society, it is considered that sex is only for procreation, and in later life, involving in sexual activity is considered as a stigma (Banerjee and Rao, 2022). Sexual intimacy should be stopped when their kids grow above puberty. It would be very embarrassing and shameful if children learned that their parents were having sexual activity at a later age. Those who involve in sexual activity in later life get a negative reaction from their family members, especially from children (Rao et al., 2015). One of the subthemes in this section also shows gender differences in experiencing the consequences for “being off-time.” Compared to Jeetu, Priyamvada has experienced much harsher negative reactions from family members and relatives for continued intimacy and subsequent pregnancy (Gewirtz-Meydan et al., 2019). Stricter and profound childbearing deadlines for women compared to men could provide insight into such gender differences (Billari et al., 2011). Another subtheme, “toll for the family,” confirms the social nature of life transitions (Neugarten and Hagestad, 1976; Settersten and Thogmartin, 2018) by extending the impact of Jeetu and Priyamvada's sexual intimacy on their immediate family. The quality of relationship among family individuals influences the overall wellbeing, and poor relationship takes toll on every individual. As off-time pregnancy was a stressor in the family due to non-acceptance of sexual intimacy at middle age, it impacted the family members' wellbeing. Patrão et al. (2018a,b) found that being married or having a support from the partner increases the self-efficacy of the elderly to deal with adverse situations. In our study, Jeetu and Priyamvada were together and supporting each other in the off-time pregnancy, which acted as a buffer against the negative experiences related to it.

Family regulates or controls the behavior of each family member and provides encouragement, support, and information (i.e., social sanctions and norms) to lead a positive and healthy life. The support from family at adverse situations works as a source of coping and provides a greater sense of self-worth and enhances self-esteem (Cohen, 2004; Patrão et al., 2018a,b, 2019; Thomas and Umberson, 2018). The significant role of the family in challenging age norms is evident in the “family came forward and fix the broken box” theme. Of the two subthemes, the “modification is done to the self” theme highlights the family's role as a primary and necessary element of social change. Boulding (2017, p. 127) discussed the role of “family as an agent of social change” and articulated the diverse roles of family: “*the family is normally thought of as an instrument for the maintenance of social stability. It is perfectly possible, however, to look at it as potentially an agent of revolutionary social change*... Amma's opposition of Jeetu and Priyamvada shared intimacy and pregnancy in their early 50s could be guided by the religious practices and ashram system. On the other hand, Nakul's rejection of his parents' intimacy and pregnancy could be perceived as a result of popular practices exercised by the majority of society (Freeman et al., 2014; Gewirtz-Meydan et al., 2019; Træen et al., 2019). The process of any social change within the existing system of social norms and practices would be initiated primarily in the family. Individuals have to start accepting the proposed change. They should muster courage and confidence to present that change positively to the society as a new version of the old existing norm. That becomes evident in the subtheme “altered the broken box with an updated one.”

### Strengths and future directions

This study has taken a very significant issue of sexuality and aging in Indian context as very few studies have attempted to explore the psycho-social consequences of social norms and practices about sexual intimacy and untimely pregnancy among middle-aged couples; also, the existing literature on sexuality and aging was more medical-oriented than social-oriented. Moreover, very few studies have explored these issues in the Indian context. Using a film as a medium to study such complex and multilayered issues of sexual intimacy, societal norms, expected behavior from older couples, and prevalent attitudes toward sexual intimacy at a later age helped to delve deeper into such topic in an ethical, and time- and money-saving manner. A film is a medium that reflects societal issues and also carries the power to influence society. The findings of this study that show the significance of family support as a mechanism or agent of social change can be utilized to develop interventions or promote practices to inculcate social change in a given society. The present study underlines the significance of age norms or social norms and highlights the role of the family in maintaining or challenging such age normative deadlines in Indian society. The step to support the family member who violated the age norms suggests two significant factors: an understanding of the change in existing social norms and the key role of the family in this process. As Zimmerman (1950, p. 22) rightly said, “*The family is more closely related to and more pervasively integrated with social change than is any other human institution*.” The findings of this study also highlight the gender differences in consequences of challenging the existing age norms. Future researchers can build on these findings with empirical research and explore in detail the facilitators and hindrances of changes in existing social norms or dominant cultural practices. Further research could extend the findings of this study by directly involving participants with their real-life experiences of challenging age norms in contemporary Indian society.

## Data availability statement

Publicly available datasets were analyzed in this study. This data can be found at: https://www.hotstar.com/in/movies/badhaai-ho/1000120365.

## Author contributions

All authors listed have made a substantial, direct, and intellectual contribution to the work and approved it for publication.

## Conflict of interest

The authors declare that the research was conducted in the absence of any commercial or financial relationships that could be construed as a potential conflict of interest.

## Publisher's note

All claims expressed in this article are solely those of the authors and do not necessarily represent those of their affiliated organizations, or those of the publisher, the editors and the reviewers. Any product that may be evaluated in this article, or claim that may be made by its manufacturer, is not guaranteed or endorsed by the publisher.
